# An In Vitro Study of Antioxidant, Anti-inflammatory, and Cytotoxic Effects of Echinacea-Mediated Zinc Oxide Nanoparticles

**DOI:** 10.7759/cureus.65354

**Published:** 2024-07-25

**Authors:** Nivedha Nedumaran, Arvina Rajasekar, Souparnika Venkatakrishnan, Hidhayathul Wajeeha

**Affiliations:** 1 Periodontics, Saveetha Dental College and Hospitals, Saveetha Institute of Medical and Technical Sciences, Saveetha University, Chennai, IND

**Keywords:** zinc oxide nanoparticles, synthesis, cytotoxic potential, antioxidant, anti-inflammatory

## Abstract

Background

Plant extracts, such as *Echinacea*, are preferred in the pharmaceutical industry for their natural availability and minimal adverse effects. *Echinacea* is known for its anti-inflammatory and other biological properties. Zinc oxide nanoparticles (ZnONPs) are cost-effective, safe, and easily synthesized, making them prominent in nanoparticle research. This study aims to determine the anti-inflammatory, cytotoxic, and antioxidant properties of ZnONPs synthesized using *Echinacea*.

Methodology

In this study, 5 mg of powdered *Echinacea* was mixed with 100 mL of distilled water, heated at 44°C until vaporization, cooled, and filtered twice. The extract was mixed with 0.1 g of zinc oxide and exposed to sunlight for two weeks for nanoparticle synthesis. After centrifugation at 3,500 rpm for eight minutes, nanoparticles were collected. Scanning electron microscope analysis was done to determine nanoparticle formation. Cytotoxicity analysis was conducted using the brine shrimp method, with surviving nauplii counted after exposure to different nanoparticle concentrations. Antioxidant activity was assessed via 2,2-diphenyl-1-picrylhydrazyl (DPPH) assay and ferric-reducing antioxidant power (FRAP) assay. Anti-inflammatory activity was assessed using membrane stabilization assay and bovine serum albumin (BSA) assay. Using SPSS Statistics Version 23 (IBM Corp., Armonk, NY, USA), the mean and standard deviation between the prepared extract and the standard were compared for all assays.

Results

In the cytotoxicity assessment, at 5 µL, the mortality of nauplii remained unchanged from the control. However, at 10 and 20 µL, a 10% increase in mortality was observed, which then stabilized at 40 and 80 µL with 20%. Regarding antioxidant activity, as nanoparticle concentration increased from 10 to 50 µL in the DPPH and FRAP assays, their effectiveness also increased accordingly. According to the anti-inflammatory assay, the membrane stabilization and BSA assay showed an increase in activity with increasing concentrations of 10 to 50 μL extract against similar concentrations of standard diclofenac sodium.

Conclusions

*Echinacea*-based ZnONPs demonstrated effective antioxidant and anti-inflammatory properties with low cytotoxicity, suggesting their potential use in future pharmaceutical or therapeutic applications.

## Introduction

Nanotechnology represents a swiftly advancing area of research utilizing biosynthetic methods and eco-friendly technologies. By applying nanostructures and nanophases to a variety of scientific domains, particularly in nanomedicine and nano-based drug delivery systems, where such particles are of great interest, nanotechnology has been demonstrated to bridge the gap between the biological and physical sciences. Nanomaterials refer to particles with extremely small dimensions on the nanoscale, whereas nanoparticles are even smaller and possess high catalytic reactivity, nonlinear optical properties, thermal conductivity, and chemical stability because of their significant surface area compared to volume [1]. Nanoparticles have specific biological, mechanical, and structural characteristics. The ability to use nanoparticles as delivery agents to encapsulate or attach therapeutic pharmaceuticals and deliver them to target tissues more accurately with a controlled release has led to the increased appreciation of nanomedicines in recent years.

Metals are preferred as nanoparticles due to their unique optical properties and they exhibit high catalytic activity, making them effective in chemical reactions. Metals can create oxides with a variety of structural geometries and characteristics, such as metallic, semiconductor, or insulating. The research of these materials’ interaction with cells was made possible by the proper nanoscale manipulation of these materials and the creation of metal oxide nanoparticles (MONPs). Despite warnings about the potentially harmful effects of MONPs, they have been widely employed in a range of industries, including healthcare. The production of MONPs typically involves costly and hazardous physical or chemical methods, utilizing antagonistic chemicals as stabilizing agents [2].

Among the array of synthesized nanoparticles, zinc oxide nanoparticles (ZnONPs) hold particular prominence because of their cost-effectiveness, safety, and simple preparation [3]. ZnONPs are a potent inorganic material of choice and have biological applications because of their remarkable biocompatibility, cost-effectiveness, and low toxicity. They hold great promise in biomedicine, particularly for their potent capability to counter excessive production of reactive oxygen species, release zinc ions, and induce cell apoptosis. Furthermore, zinc is well-known for its role in preserving the structural integrity of insulin [4]. Hence, ZnONPs were explored for managing diabetes. Additionally, zinc oxide has been extensively researched for its optical, antioxidant, wound healing, anti-inflammatory, antidiabetic, antifungal, and antibacterial properties [5].

Recent advancements have highlighted the significant recognition of utilizing green synthesis for formulating nanoparticles of metals and metallic oxides. This method involves the incorporation of herbal products containing extracts from various plant components, serving as reducing agents and popular choices during synthesis. Plant extracts are increasingly favored in pharmaceutical industries for their natural availability and minimal side effects. Certain spices are renowned for their anti-inflammatory, antioxidant, and chemoprotective properties [6-9]. Indigenous communities have traditionally used *Echinacea* species, which are native to North America. *Echinacea* is also referred to as the purple coneflower, which is a traditional herbal, natural medicine used for treating upper respiratory infections, coughs, bronchitis, common colds, and inflammatory diseases. Although many of the active chemicals have been identified in *Echinacea*, their modes of action, bioavailability, relative potency, or synergistic effects are still unknown [10]. The consensus from research suggests that *Echinacea* can indeed be efficient in reducing both the duration and severity of symptoms, albeit with specific preparations. Studies have demonstrated that the plant and its active constituents influence the phagocytic immune system [11]. Therefore, in the present research, we aimed to determine the cytotoxic, antioxidant, and anti-inflammatory properties of ZnONPs synthesized using *Echinacea*.

## Materials and methods

Preparation of the extract

**Figure 1:**
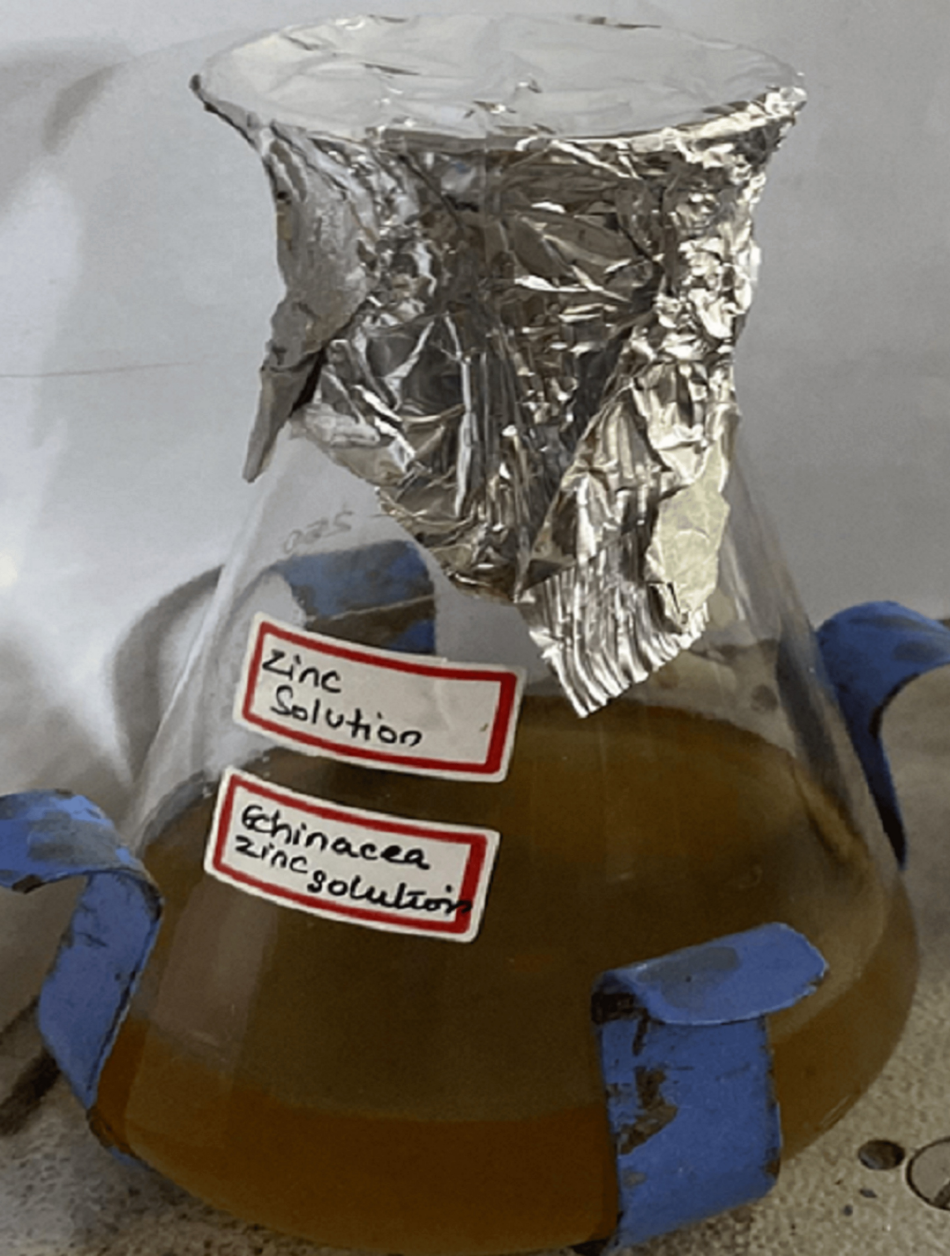
Prepared Echinacea extract.

A 5 mg sample of powdered *Echinacea* was combined with 100 mL of distilled water and the mixture was heated in a hot air oven at 44°C for two hours until vaporization occurred. The resulting extract was then cooled and subjected to filtration. This freshly prepared *Echinacea* extract was filtered once more using Whatman No. 1 filter paper. The filtered extract was collected and kept at room temperature [12], as shown in Figure 1.

Synthesis of zinc oxide nanoparticles

To synthesize ZnONPs, 0.1 g of zinc oxide was introduced into 100 mL of the filtered *Echinacea* extract. The reaction mixture was then subjected to sunlight irradiation for two weeks until a noticeable color change occurred. Following this, the final reaction mixture underwent centrifugation at 3,500 rpm for eight minutes, resulting in the collection of nanoparticles [13].

Topography analysis

Using a scanning electron microscope (JEOL JSM-IT 800, USA Inc., Peabody, MA, US), the surface morphology of the samples was examined. To prepare the material for morphological studies, it was fractured using liquid nitrogen [14]. Then, the samples were sputter-coated with platinum.

Cytotoxic analysis

Brine Shrimp Lethality Assay

The cytotoxic effect of *Echinacea*-mediated ZnONPs was evaluated with the brine shrimp method. Eggs of brine shrimp (*Artemia salina*), which were obtained locally from Aquatic Remedies in Chennai, were incubated in artificial seawater made of 40 g/L of sea salt supplemented with 6 mg/L of dried yeast and oxygenated using an aquarium pump. The nauplii were harvested by attracting them to one side of the vessel using a light source and collected using a Pasteur pipette following 48 hours of incubation in a warm environment (22°C to 29°C). The nauplii were separated from the eggs by transferring them two to three times into small beakers filled with seawater using a pipette. Subsequently, 10 nauplii were introduced into each well filled with NaCl solution, and various concentrations (ranging from 10 to 50 μL) of the *Echinacea*-mediated ZnONPs were added to each well. A control group containing only nauplii and NaCl solution was used for comparison. The extract’s lethality was evaluated by counting and recording the number of surviving nauplii in each well after the wells were left undisturbed for a full day using the following formula: number of dead nauplii/number of dead nauplii + number of live nauplii × 100 [14].

Antioxidant activity

2,2-Diphenyl-1-Picrylhydrazyl Assay

2,2-diphenyl-1-picrylhydrazyl (DPPH) assay antioxidant activity was conducted. Different concentrations (ranging from 10 μL to 50 μL) of the nanoparticles were mixed with a solution comprising 450 μL of 50 mM Tris-HCl buffer (pH 7.4) along with 1 mL of 0.1 mM DPPH in methanol followed by a 30-minute incubation period. The decrease in DPPH free radicals was assessed by the absorbance at 517 nm after incubation, with butylated hydroxytoluene (BHT) serving as the control [15]. The percentage inhibition was computed using the following formula: absorbance of the control - absorbance of the sample)/absorbance of control × 100.

Ferric-Reducing Antioxidant Power Assay

In this assay, 300 mM acetate buffer with a pH of 3.6; 2,4,6-tripyridyl-s-triazine (TPTZ) 10 mM in 40 mM HCl of molecular weight 312.34; 20 mM of FeCl_3_.6H_2_O molecular weight 270.30 were the three components used. To calculate the volume, 1 g of sodium acetate trihydrate was weighed and then added to 16 mL of glacial acetic acid and 1 L of distilled water. All three components were combined in a ratio of 10:1:1 to create the functional ferric-reducing antioxidant power (FRAP) reagent before testing. The standard ranged from 0.1 to 1.5 mM in methanol. For five minutes, distilled water (0.4 mL) and the FRAP solution (3.6 mL) were mixed and incubated at 37°C. Following the addition of this solution, 80 mL of plant extract was added and the mixture was heated to 37°C for 10 minutes. The absorbance was measured at 593 nm. The values were determined for sample solutions and five concentrations of FeSO_4_.7H_2_O (0.1, 0.4, 0.8, 1, 1.12, and 1.5 mM) to create the calibration curve [16].

Anti-inflammatory activity

Membrane Stabilization Assay

The in vitro membrane stabilization assay is a widely used technique for evaluating the ability of both natural and manufactured compounds to stabilize membranes. The purpose of this test is to determine whether a substance can maintain the integrity of the cell membrane and prevent the leakage of intracellular materials. Phosphate buffer solution centrifuge tubes, a UV-visible spectrophotometer, human red blood cells (RBCs), and different amounts of ZnONP extract (10-50 μL) in tris-HCl buffer were the materials used. Overall, 1 mL of the RBC suspension was pipetted into each centrifuge tube. Subsequently, different concentrations of ZnONP extract were introduced into each tube. The tubes were gently mixed and then incubated at 37°C for 30 minutes. The blood was then centrifuged for 10 minutes at room temperature at 1,000 rpm. The absorbance of the supernatant at 540 nm wavelength was measured with a UV-visible spectrophotometer [17]. The percent inhibition of hemolysis was calculated using the following formula: % inhibition = (OD control - OD sample)/OD control × 100.

Bovine Serum Albumin Assay

The experiment was performed with the bovine serum albumin (BSA) assay as the reagent [18]. Of the total protein in animal serum, about 60% is composed of BSA. BSA denatures and expresses antigens when heated. Five test tubes with different amounts (10-50 μL) of the produced nanoparticle were combined with 2 mL of the 1% bovine albumin fraction. To bring the pH of the reaction mixture down to 6.8, 1 N HCl was then used. The reaction mixture was incubated for 20 minutes at room temperature in a water bath. After letting the mixture cool to room temperature, the absorbance at 660 nm was measured. The standard used was diclofenac sodium in various concentrations. Percent inhibition was calculated using the following formula: % inhibition = (control OD - sample OD)/control OD × 100.

Statistical analysis

The statistical analysis was done using SPSS Statistics Version 23.0 (IBM Corp., Armonk, NY, USA). The mean and standard deviation were compared between the prepared extract and the standard for all the assays.

## Results

Topographic analysis

**Figure 2:**
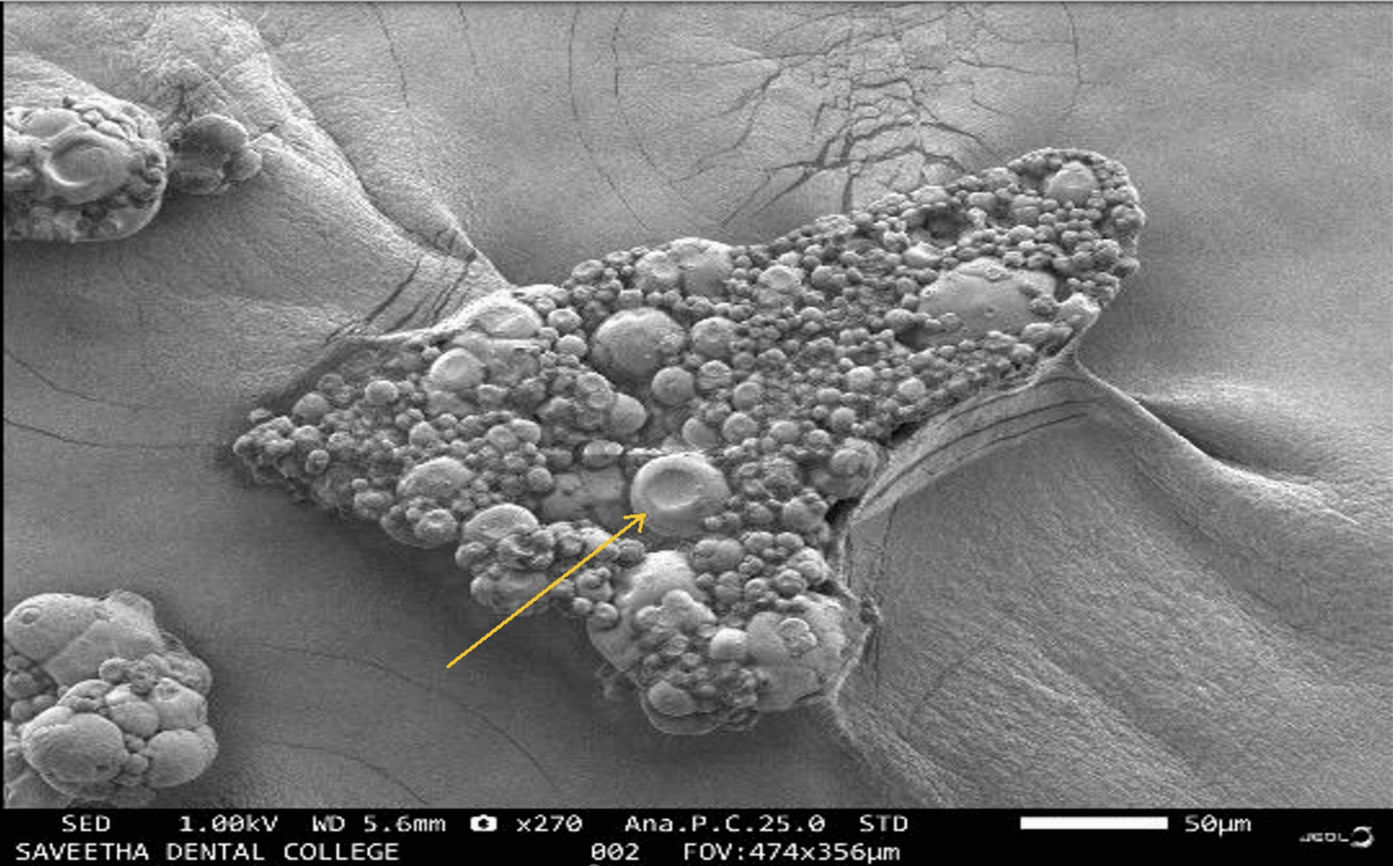
Scanning electron microscope image showing Echinacea-mediated zinc oxide nanoparticles at 270×.

The topographic analysis was done using scanning electron microscopy. The image showed the presence of *Echinacea*-mediated ZnONPs under 270× magnification (Figure 2).

Cytotoxic effect

**Figure 3:**
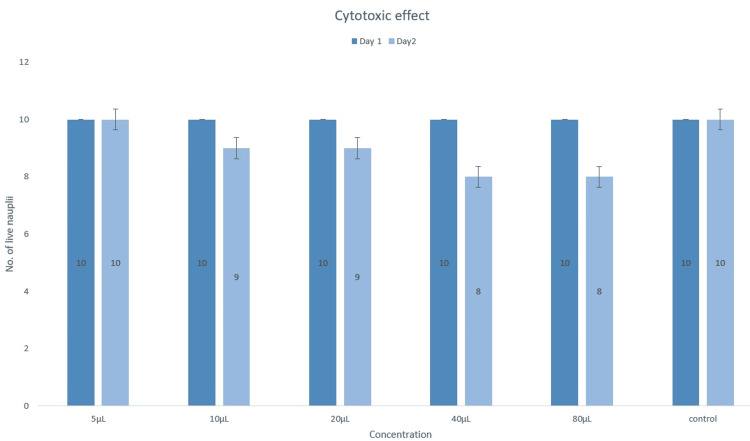
Cytotoxic effect showing the lethality of the nauplii at different concentrations using the brine shrimp lethality assay.

Figure 3 illustrates the lethality of the nauplii at various concentrations (5-80 µL). Observations were made over two days across concentration levels of 5 μL, 10 μL, 20 μL, 40 μL, and 80 μL. At a concentration of 5 μL, the mortality rate remained unchanged compared to the control. However, a 10% increase in mortality rate from the control was noted at concentrations of 10 μL and 20 μL. Subsequently, at higher concentrations of 40 μL and 80 μL, the mortality rate doubled to 20%, but then appeared to stabilize, suggesting a diminishing effect at higher concentrations.

Antioxidant effect

**Figure 4:**
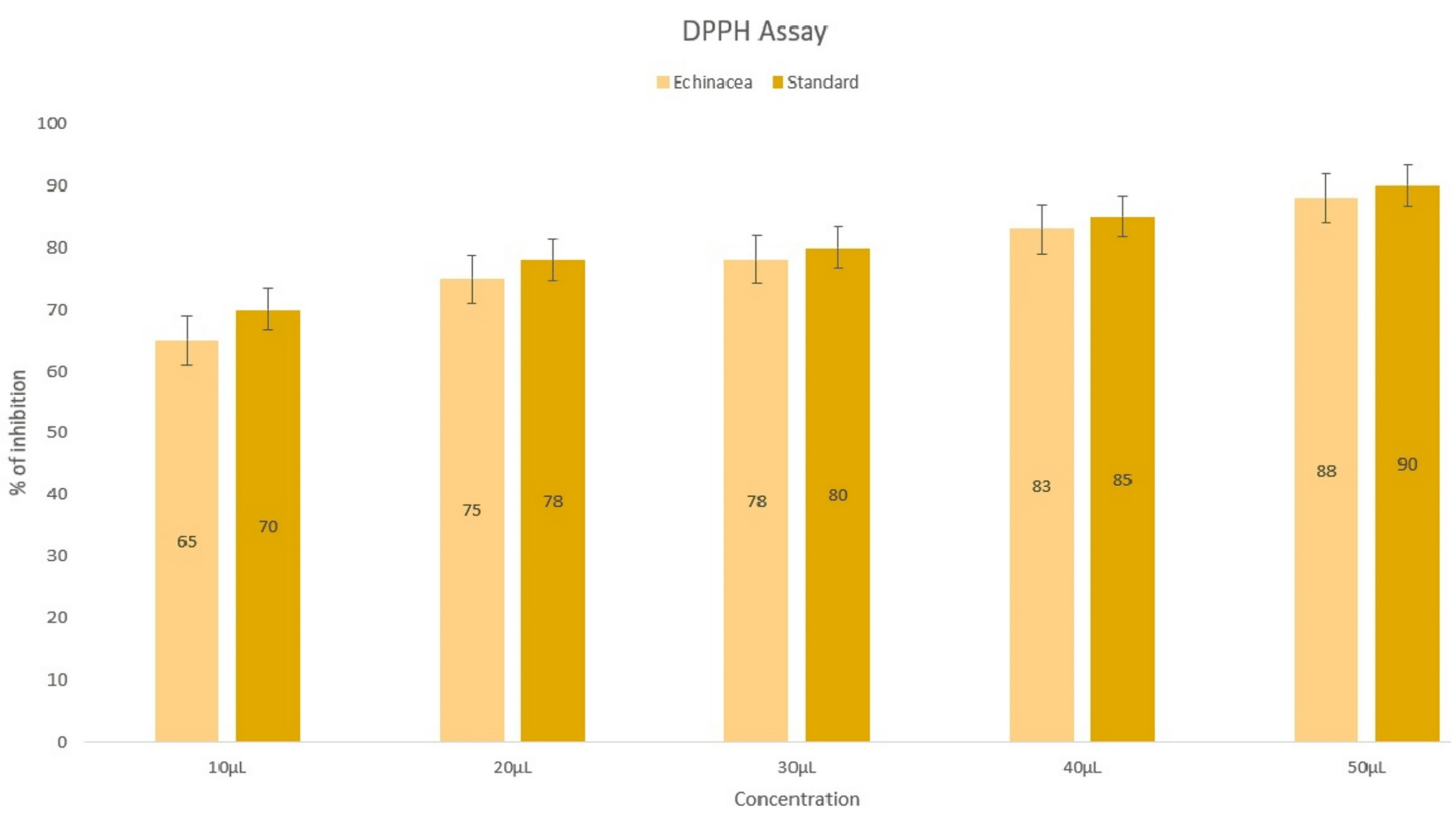
Antioxidant activity of zinc oxide nanoparticles synthesized using Echinacea using the 2,2-diphenyl-1-picrylhydrazyl assay.

According to the DPPH assay, the sample was originally tested with 10 μL; subsequently, the concentration was increased to 20 μL, 30 μL, 40 μL, and 50 μL. The antioxidant activity of the nanoparticles increased on a scale based on the DPPH assay because their concentration increased by 10 μL with each test when compared to the standard BHT (Figure 4).

**Figure 5:**
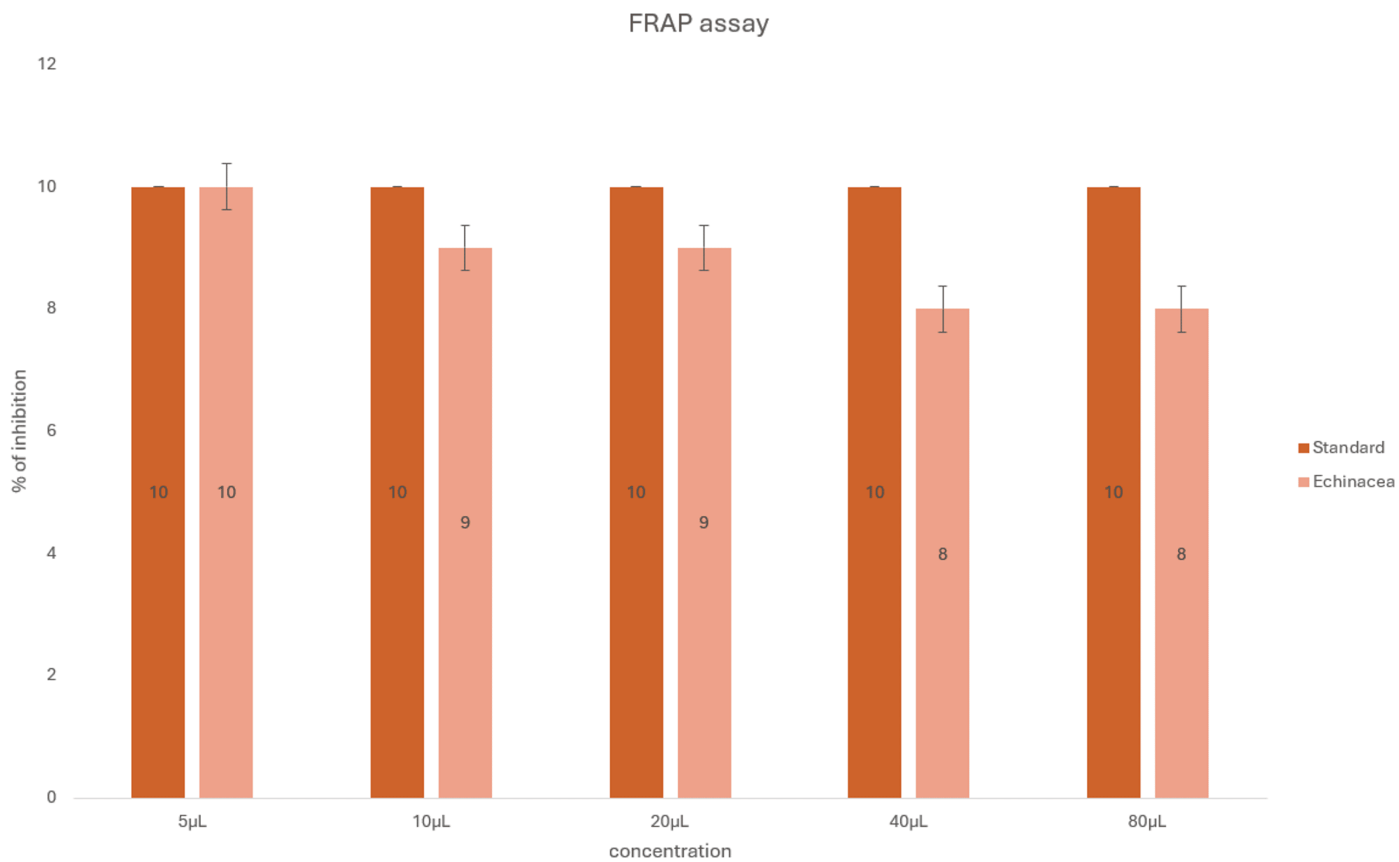
Antioxidant activity of zinc oxide nanoparticles synthesized using Echinacea using the ferric-reducing antioxidant power assay.

To obtain unbiased results, the samples were tested using another assay. According to the FRAP assay (Figure 5), the antioxidant levels increased with the increase in the concentration of the extract compared to the standard with a mean difference of <5% in terms of the standard and the sample tested.

Anti-inflammatory activity

**Figure 6:**
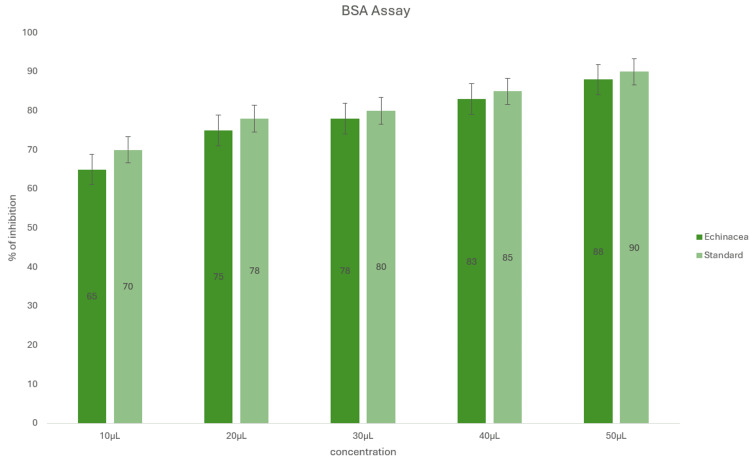
Anti-inflammatory activity of zinc oxide nanoparticles synthesized using Echinacea using the bovine serum albumin assay.

In Figure 6, according to the bovine serum assay, the albumin denaturation results showed that ZnONPs had a significant anti-inflammatory effect. At 50 μL, the highest inhibition was over 77%, which is comparable to the standard drug, diclofenac sodium. The produced nanoparticle was nearly identical to the reference medication in each concentration that was employed. As concentration increased from 10 μL to 50 μL, ZnONPs utilizing *Echinacea* exhibited greater anti-inflammatory effects.

**Figure 7:**
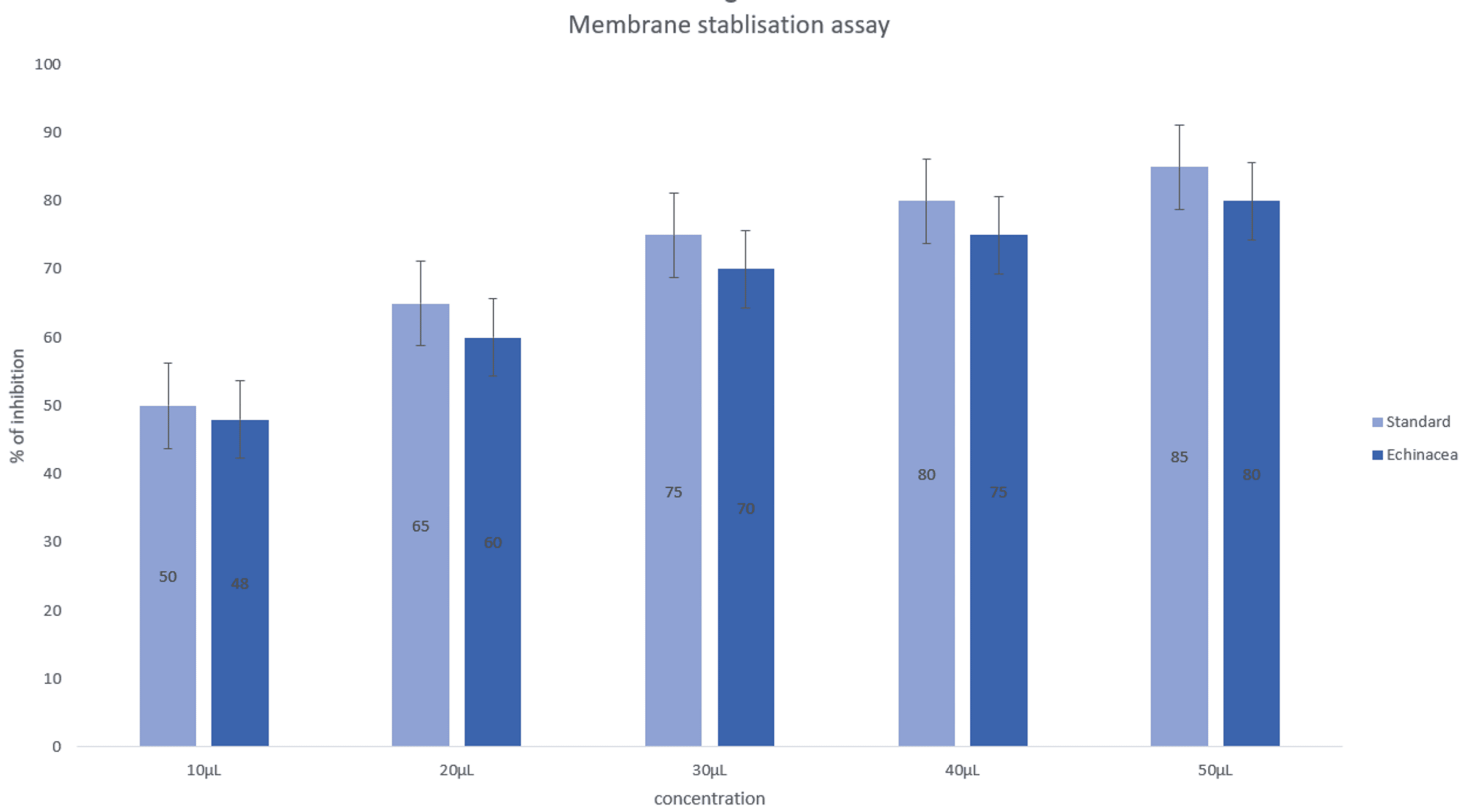
Anti-inflammatory activity of zinc oxide nanoparticles synthesized using Echinacea using the membrane stabilization assay.

The activity was tested using another assay, namely, the membrane stabilization assay (Figure 7). There was an increase in activity with increasing concentrations of 10 μL, 20 μL, 30 μL, 40 μL, and 50 μL extract against similar concentrations of standard diclofenac sodium.

## Discussion

Phytotherapy is a way of treating, relieving, and preventing diseases by employing complete medicinal plants or their components. Today, many scientific disciplines use chemical, biodynamic, and pharmacological methods to justify the use of medicinal plants and preparations, as well as their advantage over chemical preparations for the treatment of various diseases, ailments, and disorders affecting the human body. In their comprehensive review, Caruso et al. [19] proposed the efficacy of *Echinacea* in treating the common cold. Additionally, a systematic review indicated that preparations derived from the aerial parts of *Echinacea* could be effective for early treatment of the common cold in adults, though results were not entirely consistent [20]. A meta-analysis revealed a 58% reduction in the odds of developing the common cold and a 1.4-day decrease in cold duration with *Echinacea* usage, supporting its benefit in reducing both incidence and duration [21].

In this study, *Echinacea*-mediated ZnONPs exhibited potent antioxidant activity. Kasote et al. [22] conducted a study that demonstrated the physiology and redox biology of plant species to improve our understanding of plant antioxidants as therapeutic entities. They highlighted that the antioxidant potential of the *Echinacea* species is due to the presence of total phenolic content. These plants act as strong antioxidants with significant reducing capacity, facilitating nanoparticle synthesis. The electron-donating ability of phenolic compounds aids in reducing zinc ions to nano-sized particles by transferring electrons, thereby facilitating nanoparticle formation. Phenolic compounds are well-documented for their direct contribution to antioxidative activity owing to their redox properties, which enable them to act as reducing agents, singlet oxygen quenchers, and hydrogen donors. Similarly, Stanisavljević et al. [23] evaluated the antioxidant and antimicrobial activities of *Echinacea purpurea* L. extracts, demonstrating higher amounts of flavonoids and phenolic compounds, along with significant DPPH scavenging activity and growth inhibition of certain microorganisms. Our study findings are in accordance with the previous studies.

The brine shrimp lethality bio-assay (BSLB) has been widely employed to assess toxicity in crude extracts and isolated compounds due to its simplicity and efficiency, allowing for the screening of numerous samples during drug discovery [24]. The present study used the BSLB assay and observed that the mortality of the nauplii increased with the increasing concentration of *Echinacea* till it attained a threshold level. Yazdanian et al. [25] evaluated the cytotoxic effect of *Echinacea* using the MTT assay and showed a significant reduction in toxicity up to the threshold concentration of 0.625 mg/mL. Similar results were obtained in another in vitro study by Tsai et al. [26]. In the present study, we also observed that the anti-inflammatory activity of synthesized *Echinacea*-mediated ZnONPs was identical to the standard drug, diclofenac sodium. These findings align with the results obtained by Mohamed et al. [27] who demonstrated the anti-inflammatory properties of magnesium-doped ZnO synthesized from *Ficus religiosa* leaves. Furthermore, green synthesis-mediated ZnONPs offer anti-inflammatory properties as they contain bioactive compounds. Additionally, the biocompatibility of naturally synthesized nanoparticles is higher, reducing the risk of adverse immune reactions. This method also ensures that the nanoparticles are free from harmful chemical residues, making them safer for medical and therapeutic applications [28].

Furthermore, Gecer et al. [29] produced silver nanoparticles (AgNPs) from the aerial portion of *Echinacea*. X-ray diffraction, Fourier transform infrared, UV-visible, and scanning electron microscopy spectroscopy were used to gain insights into the produced AgNPs. Using DPPH and reducing power experiments, the antioxidant activity of AgNPs was investigated. AgNPs and the extract exhibited good antioxidant activity, which may have applications in both food and medicine. The findings coincided with our study findings, as the anti-inflammatory characteristic of ZnONPs utilizing *Echinacea* increased as the concentrations increased from 10 μL to 50 μL. Based on the low cytotoxicity observed in the BSLB, biologically produced ZnONPs offer a preferable, cost-effective alternative to physical and chemical methods. They are rapid, clean, and environmentally friendly, with potential applications in pharmaceuticals, agriculture, and textiles [30].

However, the in vitro study design affects the reliability and applicability of the findings. Therefore, to validate our findings, future research should include in vivo studies and advanced in vitro models that better mimic the physiological environment. Additionally, isolating each bioactive compound in *Echinacea* and evaluating their individual effects will help identify the specific components responsible for the observed therapeutic benefits.

## Conclusions

The present study demonstrated that ZnONPs can be efficiently synthesized via an eco-friendly method using *Echinacea* extract, highlighting the potential of green synthesis over traditional physical and chemical methods. These ZnONPs can be formulated into various products such as pastes, gels, and oils, capitalizing on their enhanced anti-inflammatory and antioxidant properties for medical applications. The data suggest that *Echinacea*-mediated ZnONPs can be scaled up for large-scale production and utilized for targeted drug delivery due to their low cytotoxicity and potent anti-inflammatory activity. Future research should focus on optimizing formulations for clinical use and exploring their efficacy in vivo.
